# Comparative Analysis of the Venom Proteins from Two Eupelmid Egg Parasitoids *Anastatus japonicus* and *Mesocomys trabalae*

**DOI:** 10.3390/biology12050700

**Published:** 2023-05-10

**Authors:** Qian-Yu Zhao, Xu Chen, Run-Zhi Wang, Yong-Ming Chen, Lian-Sheng Zang

**Affiliations:** 1 National Key Laboratory of Green Pesticide, Guizhou University, Guiyang 550025, China; 2 Key Laboratory of Green Pesticide and Agricultural Bioengineering, Ministry of Education, Guizhou University, Guiyang 550025, China

**Keywords:** parasitic wasp, Eupelmidae, *Caligula japonica*, venom proteins

## Abstract

**Simple Summary:**

Research on the composition of egg parasitoid venom is very limited. In this study, we used a combination of transcriptomic and proteomic approaches to identify the protein fractions of the venom in both eupelmid egg parasitoids, *Anastatus japonicus* and *Mesocomys trabalae*. We identified 3422 up-regulated venom gland genes in *M. trabalae* and 3709 in *A. japonicus* and analyzed their functions comparatively. By proteome sequencing, we identified 956 potential venom proteins in the venom pouch of *M. trabalae*, of which 186 were contained in up-regulated genes simultaneously. A total of 766 proteins were detected in the venom of *A. japonicus*, of which 128 venom proteins were highly expressed in the venom glands. The results of functional analysis for these identified venom proteins indicated that the venom proteins in *M. trabalae* are well known but not in *A. japonicus,* which may be related to the host range. These results will provide a theoretical basis for studying the function of egg parasitoid venom and its parasitic mechanism.

**Abstract:**

Parasitic wasps are abundant and diverse Hymenoptera insects that lay their eggs inside or on the external surface of the host and inject venom into the host to create a more favorable environment for the larvae to survive and regulate the host’s immunity, metabolism, and development. But research on the composition of egg parasitoid venom is very limited. In this study, we used a combination of transcriptomic and proteomic approaches to identify the protein fractions of the venom in both eupelmid egg parasitoids, *Anastatus japonicus* and *Mesocomys trabalae*. We identified 3422 up-regulated venom gland genes (UVGs) in *M. trabalae* and 3709 in *A. japonicus* and analyzed their functions comparatively. By proteome sequencing, we identified 956 potential venom proteins in the venom pouch of *M. trabalae*, of which 186 were contained in UVGs simultaneously. A total of 766 proteins were detected in the venom of *A. japonicus*, of which 128 venom proteins were highly expressed in the venom glands. At the same time, the functional analysis of these identified venom proteins was carried out separately. We found the venom proteins in *M. trabalae* are well known but not in *A. japonicus,* which may be related to the host range. In conclusion, identifying venom proteins in both egg parasitoid species provides a database for studying the function of egg parasitoid venom and its parasitic mechanism.

## 1. Introduction

Parasitic wasps, the most abundant and diverse insect species in Hymenoptera, are critical natural control factors for insects and effective biocontrol agents [1]. Parasitic wasps usually carry parasitic factors released into the host during egg laying or embryo and larval development to regulate critical physiological processes in the host to ensure successful parasitism of the host pest and normal development of its offspring [2,3,4]. These parasitic factors include polydnavirus (PDV), venom, virus-like particles (VLP), female-carrying factors such as ovarian proteins, and parasitic factors carried or released by embryos or larvae such as teratocytes [5,6,7,8,9,10,11]. And the venom of parasitic wasps is a crucial factor affecting the success of their parasitism [12]. The venom of parasitoids is a complex mixture of active substances with unknown biological functions that can be used to modulate host behavior, immunity, development, and metabolism [13]. The presence of venom is conservative among parasitic wasps. Venom proteins are produced in special glands of Hymenoptera parasitoids associated with the female reproductive system [14].

In parasitoids, various components are introduced into the host when they oviposit and facilitate the development of their progeny, including venom [15]. Traditionally, venom has been defined as a toxic fluid that inflicts sudden death or paralysis on the host or prey. They have been recognized as a rich source of biologically active compounds. In particular, the venom of snakes, scorpions, spiders, and bees has received significant attention from the biological community, and these species have been intensively studied. Venom proteins of medical value have been identified and characterized by combining transcriptomic, proteomic, and peptide-led techniques [16,17,18,19,20,21]. Some studies have been conducted on several parasitoid venom components [13,22,23,24,25,26,27,28,29,30,31,32]. Still, these studies on the function of parasitoid venom have mainly focused on larval and pupal parasitoids, and our understanding of the venom protein fractions and function of egg parasitoids is still limited.

*Anastatus japonicus* and *Mesocomys trabalae* are two egg parasitoids in Eupelmidae, Hymenoptera. They can be used as natural enemy insects to control a vital forestry pest, the Japanese Giant Silkworm, *Caligula japonica* (Lepidoptera: Saturniidae). *A. japonicus* has a wide host range and can parasitize Hemiptera insects, such as *Erthesina fullo* and *Halyomorpha picu*. *M. trabalae* does not parasitize Hemiptera insects, but it is the dominant species parasitizing *C. japonica* eggs in the natural field [33]. It has been reported that *A. japonicus* has no parasitic factors other than the venom received in the reproductive organs [34]. Its venom gland is similar to that of the endoparasitic wasp *Pteromalus puparum* (Hymenoptera: Pteromalidae) [35], and the venom of *P. puparum* has been reported to cause significant changes in the total number of host blood cells, disrupt the cytoskeleton, alter the behavior of blood cells such as stretching, adsorption, and aggregation, and affect the cystic host response to parasitoid eggs, thereby suppressing host cellular immunity [36]. Similarly, the venom of the parasitic wasp *Leptopolina boulardi* actively suppresses the host’s immune response [37].

As one of the weapons of successful parasitism in parasitic wasps, venom plays an essential role in the evolution of the host range of both parasitoids, and it has been shown that venom proteins in parasitic wasps are rapidly evolving protein taxa, with both conserved proteins and a large number of specific proteins between different parasitic wasps [38]. Humoral immunity mainly includes hemolymph nigrosis, synthesis of antimicrobial peptides, and protease and association reactions [39,40].

**Figure 1:**
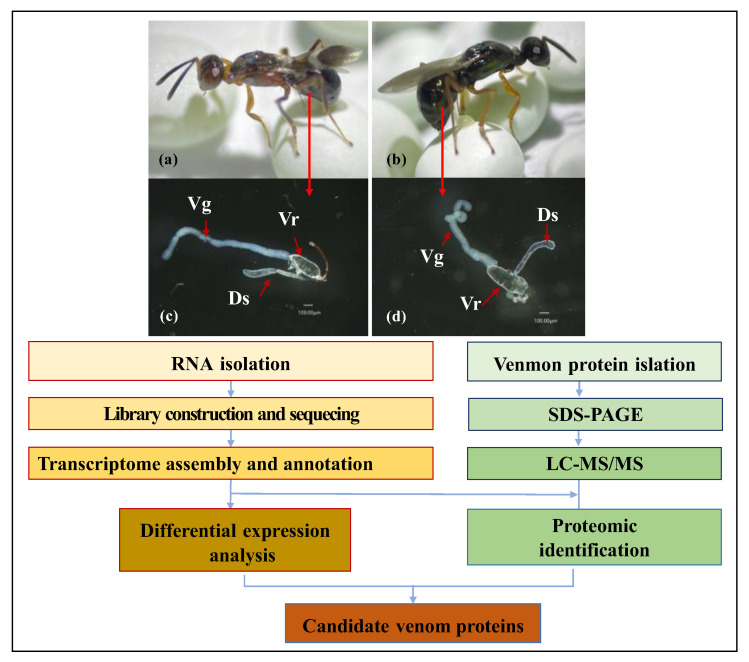
Schematic representation of combined proteomic and transcriptomic analyses to identify putative venom proteins in *Anastatus japonicus* and *Mesocomys trabalae*. (**a**): female adult of *A. japonicus*; (**b**): female adult of *M. trabalae*; (**c**): venom organs of *A. japonicus*; (**d**) venom organs of *M. trabalae*; Vr: venom reservoir; Vg: venom gland; Ds: duodenal gland.

This study used a combined transcriptomic and proteomic approach to identify and sequence the venom glands and venom of two egg parasitoids, *A. japonicus* and *M. trabalae* (Figure 1), and annotate and analyze their functions. Identifying these two egg parasitoids’ venom proteins will provide a database for future study of the function of egg parasitoids’ venom and its parasitic mechanism.

## 2. Materials and Methods

### 2.1. Insects Rearing

The egg parasitoids, *A. japonicus* and *M. trabalae,* were collected from the field in Kang County, Gansu Province (105–106° E, 32.9–33.7° N) in August 2018 [41] and bred in the Key Laboratory of Green Pesticide and Agricultural Bioengineering of the Ministry of Education, Guizhou University. The parasitoids used the eggs of *Antheraea pernyi* as alternative hosts. They were incubated in a light and constant temperature incubator at 25 ± 5 °C, photoperiod 14 L:10D, and 70 ± 5% humidity for 26–28 days and then fed with 20% honey water to ensure their continued growth and development after emergence.

### 2.2. Venom Glands Collection

Two hundred females of *A. japonicus* and *M. trabalae* emerged over 6–7 days, were fully mated, and were used to set up three replicates, respectively. The venom glands and the parasitoid bodies without venom glands were placed in Trizol (Ambion, Foster City, CA, USA), frozen in liquid nitrogen, and stored at −80 °C in a refrigerator.

### 2.3. RNA Extraction and cDNA Library Preparation

Total RNA was extracted from the venom glands and the residues with Trizol reagent, and the total amount and quality of RNA were measured by NanoDrop One/Onec2000 (Thermo Fisher Scientific Inc., Waltham, MA, USA). The integrity of the RNA was then checked precisely with a bioanalyzer (Thermo Fisher Scientific Inc., USA).

The cDNA library construction and sequencing were performed by Beijing Nuohe Zhiyuan Technology Co., Ltd. (Beijing, China). The cDNA libraries were constructed for sequencing using the NEBNext^®^ Ultra™ RNA Library Prep Kit for Illumina^®^ (NEB, Ipswich, MA, USA) kit. The extracted RNA was enriched with mRNA with polyA tails from 20 μg of total RNA by poly-T oligo-attached magnetic beads to remove ribosomal RNA, resulting in mRNA. The mRNA was fragmented at high temperatures using divalent cations in Illumina’s uniqueNEB Fragmentation Buffer. The first strand of cDNA was synthesized in the M-MuLV Reverse Transcriptase System, followed by degradation of the RNA strand with RNaseH and synthesis of the second strand of cDNA with dNTPs under the DNA polymerase I system. The purified double-stranded cDNA was end-repaired, A-tailed, and ligated with sequencing connectors. cDNAs of about 250–300 bp were screened with AMPure XP beads, PCR amplified, and the products were purified again using AMPure XP beads to finally obtain libraries. The libraries were initially quantified using a Qubit2.0 fluorometer, diluted to 1.5 ng/uL, and then the insert size of the libraries was checked using an Agilent 2100 bioanalyzer. After the insert size met expectations, the effective concentration of the libraries was accurately quantified by qRT-PCR. After the insert size met expectations, qRT-PCR was performed to accurately quantify the effective concentration of the library (the effective concentration of the zasasp library was higher than 2 nM) to ensure the quality of the library.

### 2.4. RNA-Seq and Data Analysis

After passing the library inspection, the transcriptomes of the venom glands and residues of the two wasps were sequenced using the Illumina NovaSeq 6000 (Illumina, San Diego, CA, USA) sequencing platform. We filtered the raw data for adapter sequences, ploy-N, and low-quality reads and then checked the sequencing error rate and GC% content distribution to obtain clean reads for subsequent analysis.

Clean reads were quickly and accurately compared to the reference genome (unpublished data) using HISAT2 software to obtain information on the reading positioning on the reference genome [42]. The number of reads covered by each gene (including newly predicted genes) from the start to the end range is thus counted based on the position information of the genes’ alignment on the reference genome. This analysis was performed using the featureCounts tool in the subread software. We performed a quantitative analysis of gene expression levels for each venom gland and residual sample separately and then combined them to obtain the expression matrix for all samples. Differential gene expression signature analysis was performed using DESeq2 software [43], and the screening criteria for differential genes were |log2(FoldChange)| ≧ 1 and padj ≦ 0.05. We used clusterProfiler software to perform GO functional enrichment analysis of the differential gene sets between venom glands and remnants and KEGG pathway enrichment analysis. We also compared the functional annotations of genes significantly up-regulated in the venom glands (UVGs) of the parasitoids *A. japonicus* and *M. trabalae*.

### 2.5. Venom Protein Collection

After the venom gland synthesizes, venom proteins will be stored in its venom sacs. So, we dissected venom sacs to obtain the venom. Three replicates of 400 females of both parasitoid species were taken, respectively. The venom sacs were dissected and separated from the end of the abdomen and washed three times in sterile 1× Pringle’s phosphate-buffered saline buffer (1× PBS) (Biosharp, Hefei, China). The venom sacs were punctured with the tip of dissecting forceps and dissolved in 1× PBS buffer and protease inhibitor (TransGen Biotech, Beijing, China), centrifuged at 12,000 g for 15 min at 4 °C. The supernatant was collected, frozen in liquid nitrogen, and stored at −80 °C for proteome sequencing.

### 2.6. SDS-PAGE Electrophoresis

We used the Bradford Protein Quantification Kit (Bio-Rad, Hercules, CA, USA) to prepare the BSA standard protein solution according to the instructions. The absorbance of the standard protein solution was used to draw a standard curve and calculate the protein concentration of the sample to be tested. After the electrophoresis, the samples were stained with Thomas Brilliant Blue R-250 (Amresco, Solon, OH, USA) and decolorized until the bands were clear.

### 2.7. Enzymatic Digestion of Venom Proteins and Identification by LC-MS/MS Mass Spectrometry

One hundred and twenty microgram venom protein samples of two parasitoids were taken, respectively, and the volume of protein solution was added to make up to 100 μL and mixed with three μL 1 μg/μL-trypsin and 500 μL 100 mM-TEAB buffer, mixed well, and digested overnight at 37 °C. An equal volume of 1% formic acid was added to the mixture, mixed well, and centrifuged at 12,000 g for 5 min at room temperature. Then, the supernatant was slowly passed through the C18 desalting column.

Mobile phases A (100% water, 0.1% formic acid) and B (80% acetonitrile, 0.1% formic acid) were prepared in advance. The lyophilized powder was dissolved in 10 µL of liquid A, centrifuged at 15,000 rpm for 20 min at 4 °C, and 1 µg of the supernatant was sampled for liquid chromatography. The EASY-nLCTM 1200 nano UHPLC system (Thermo Fisher Scientific Inc., USA) with a homemade pre-column (2 cm × 75 μm, 3 μm) and a homemade analytical column (15 cm × 150 μm, 1.9 μm) was used in this study. The Q ExactiveTM series mass spectrometer (Thermo Fisher Scientific Inc., USA) with a Nanospray Flex™ (ESI) ion source was used. The ion spray voltage was set at 2.3 kV. The ion transfer tube temperature was set at 320 °C. The mass spectrum was acquired in a data-dependent mode. The full scan range of the mass spectrum was 350–1500 *m*/*z*. The resolution of the primary mass spectrum was set at 60,000 (200 *m*/*z*). The parent ion with ion intensity TOP 20 (40) in the full scan was selected and fragmented by the high-energy collisional cleavage (HCD) method for secondary mass spectrometry with a resolution of 15,000 (200 *m*/*z*) and a maximum C-trap capacity of 5 × 10^4^. The maximum injection time of the C-trap was 45 ms. The collision energy of peptide fragmentation was set to 27%. The threshold intensity was 2.2 × 10^4^ with a 20s dynamic exclusion range to generate the raw data for mass spectrometry detection. The raw data were directly imported into Proteome Discoverer v3.0 software (Thermo Fisher Scientific Inc., USA) for searching the database and quantifying spectral peptides and proteins.

### 2.8. Transcriptomic and Proteomic Data Analysis

We combined transcriptome and proteome methods to identify the venom of both parasitoid species. The proteins significantly up-regulated in the venom glands and detected in the venom proteome were used as candidate venom proteins. The insect venom and online analysis database, iVenomDB [44], were used to identify venom proteins, annotate them, and classify protein family functions. GraphPad Prism v9 uses statistical analysis and the production of pictures.

## 3. Results

### 3.1. Transcriptome Analysis

**Figure 2:**
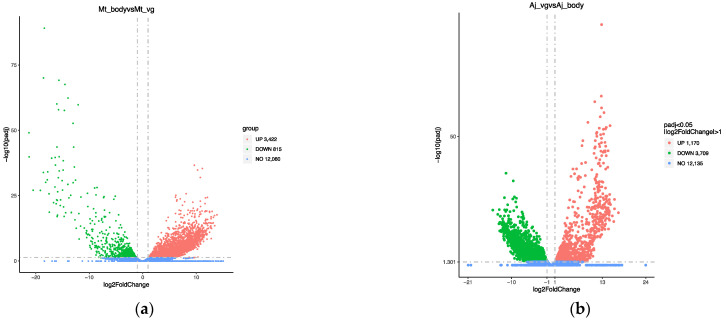
Volcano plot of differentially expressed genes of *Mesocomys trabalae* (**a**) and *Anastatus japonicus* (**b**).

The venom gland serves as the organ of venom protein synthesis, so identifying proteins requires ensuring that the venom protein gene is highly expressed in the venom gland. Through transcriptome sequencing, we obtained a total of 12 transcripts (Table 1). Briefly, 84.84 Gb of raw data were obtained from *M. trabalae*, and the proportion of bases with quality values greater than 30 to the total bases (Q30) ranged from 91.22–92.28%. The clean data obtained from sequencing were compared to the reference genome to obtain information on the positioning of reads on the reference genome. An average of 94% of the reads of the *M. trabalae* transcriptome could be compared to the reference genome, and 22,691 genes were compared in the annotation information of the genome. According to the differential gene screening criteria of |log2(FoldChange)| ≧ 1, and padj ≦ 0.05, the total number of differentially expressed genes between bodies without venom glands and venom glands of *M. trabalae* was 4237, among which 3422 highly expressed genes were identified in the venom gland of *M. trabalae* (Figure 2a). The transcriptome sequencing of *A. japonicus* resulted in 86.63 Gb of data and Q30 ranging from 90.98–93.68%. On average, 93% of the reads of the *A. japonicus* transcriptome could be compared to the reference genome, and 20,860 genes were compared in the annotation information of the genome. Furthermore, 4879 differentially expressed genes were found between the wasp bodies without venom glands and venom glands, of which 3709 were UVGs (Figure 2b). UVGs as potential candidates for venom proteins need to be further identified in combination with venom proteomic data.

### 3.2. Functional Annotation of UVGs

**Figure 3:**
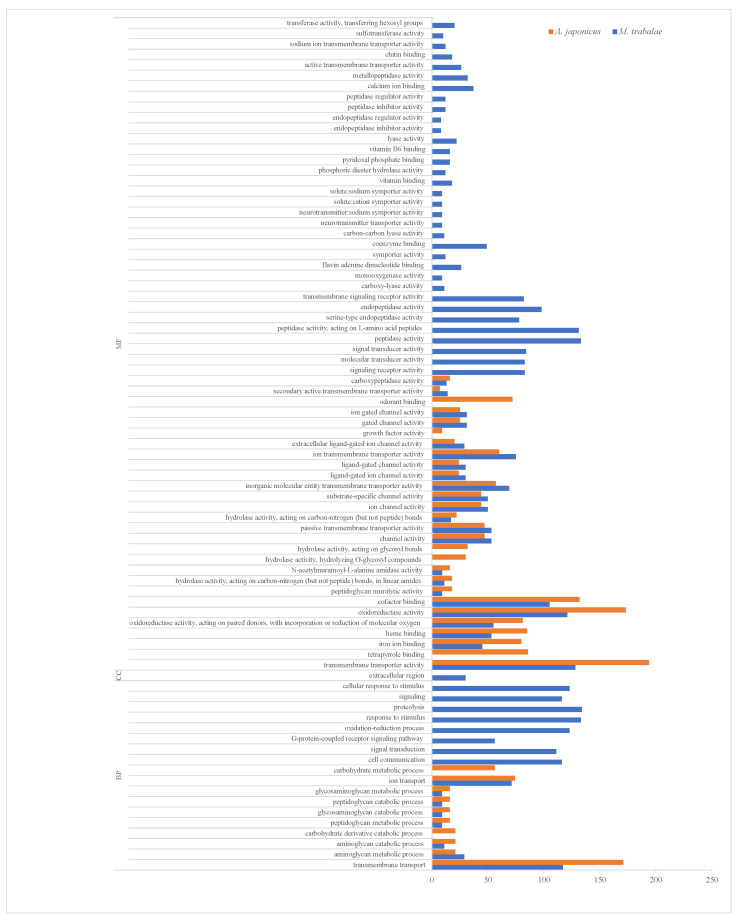
The most enriched GO terms of up-regulated genes (UVGs) in *Mesocomys trabalae* and *Anastatus japonicus*. MF: molecular function; CC: cellular component; BP: biological process.

**Figure 4:**
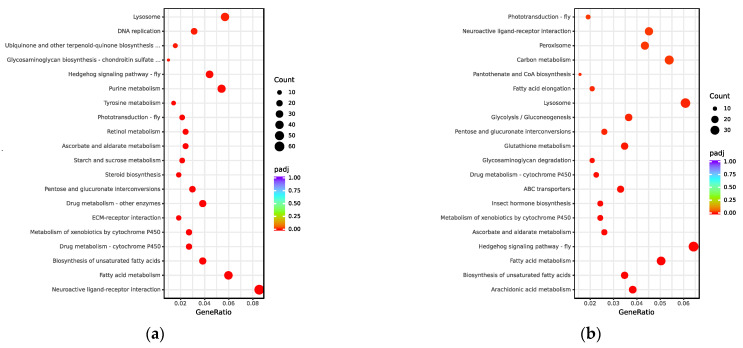
The most enriched KEGG pathways of UVGs of *Mesocomys trabalae* (**a**) and *Anastatus japonicus* (**b**).

To investigate the functions of the UVGs in *M. trabalae* and *A. japonicus*, they were subjected to GO functional enrichment analysis (Figure 3). The results showed that the functional distribution of UVGs between the two wasps was divergent. The UVGs of *M. trabalae* were enriched for 79 functions, mostly focused on molecular functions, with 2717 genes. And the number of genes with transporter activity functions was the highest, with 141, followed by genes related to enzyme activity. A total of 1176 genes were annotated in the classification of biological processes, of which the most significant number of genes related to protein hydrolase activity was 134. This was followed by stimulus response-related genes with 133. Accordingly, it is speculated that these UVGs may be involved in regulating the immune and physiological metabolism of the host. Among both parasitoid species, the cellular components were annotated rare genes, with only *M. trabalae* annotated 30 genes and UVGs of *A. japonicus* not annotated to this functional classification. In addition, 543 genes were enriched to 26 pathways in the KEGG pathway enrichment analysis for UVGs in *M. trabalae*. Among the top 20 pathways, the highest percentage was neuroactive ligand-receptor interaction (60), followed by fatty acid metabolism (42) and lysosome (38), respectively (Figure 4a).

The UVGs of *A. japonicus* were annotated in the GO database with 1916 genes in 38 functional classifications (Figure 3). As with *M. trabalae*, most of these genes are distributed in molecular functional classifications (27 in total), for 1488 genes. The most significant number of genes with transmembrane transporter activity function was 194, followed by oxidoreductase activity (173). In the biological process classification, 428 genes were annotated into ten pathways, and the functional classification with the highest number of genes was transmembrane transport, which contained 171 genes. In the KEGG database, there are 555 genes enriched to a total of 31 pathways. The top three most numerous were the hedgehog signaling pathway, biosynthesis of cofactors, and lysosome, with 37, 36, and 35 genes, respectively (Figure 4b). The UVGs may include genes encoding venom proteins and other metabolic pathways related to their synthesis pathways. Thus, UVGs contain a larger number of genes and are enriched to a larger number of pathways. However, the results of this section indicate that the UVGs of *M. trabalae* are not only more numerous than those of *A. japonicus* but also more functionally abundant.

### 3.3. SDS-PAGE and LC-MS/MS Analysis

**Figure 5:**
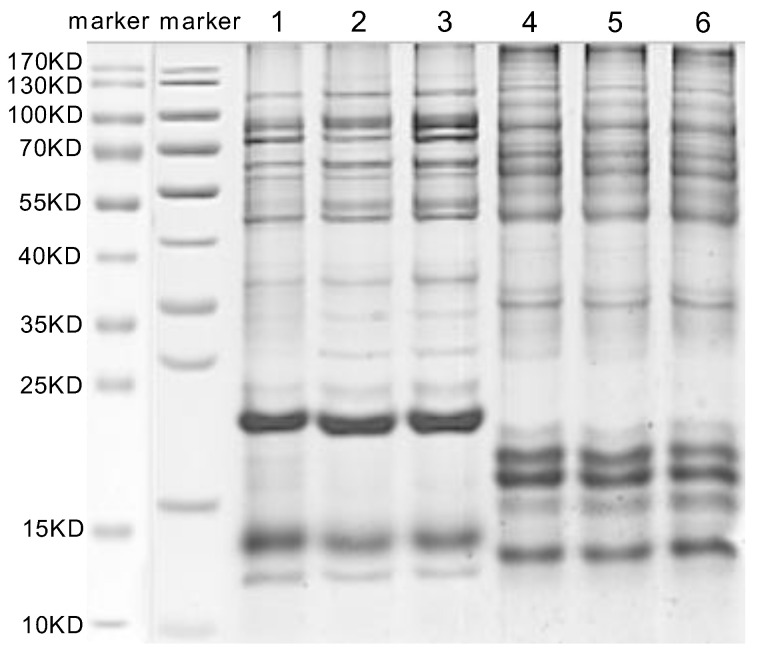
The SDS-PAGE analysis of venom protein in *Mesocomys trabalae* (**1–3**) and *Anastatus japonicus* (**4**–**6**). Marker: molecular weight marker.

**Figure 6:**
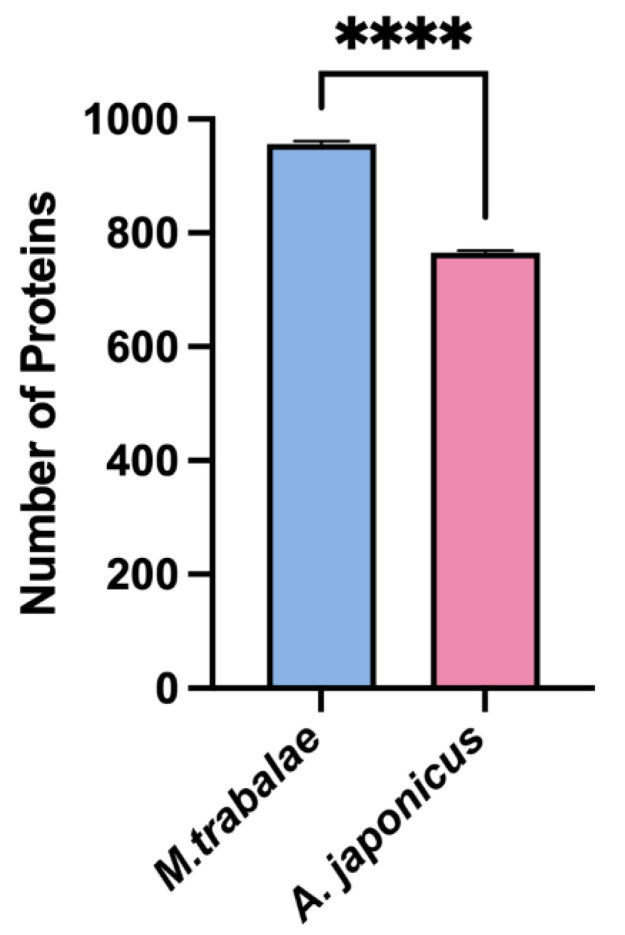
Total number of proteins in venom reservoir of *Mesocomys trabalae* and *Anastatus japonicus* identified by LC-MS/MS. Asterisks (****) above the standard error bars indicate significant differences (*t*-test, *p* < 0.01).

The *M. trabalae* and *A. japonicus* venom proteins were separated by SDS-PAGE and found to be distributed in molecular sizes from less than 15 KD to greater than 130 KD (Figure 5). The venom protein of *M. trabalae* had a broader band at 20 KD, indicating that the protein size was more abundant at 20 KD. The bands of *A. japonicus* had three more distinct bands at less than 20 KD, indicating a higher protein content at the corresponding sizes of these three bands. LC-MS/MS detection of venom proteome raw files and database search using Proteome Discoverer software showed that the number of candidate venom proteins identified in the *M. trabalae* venom samples (average of 956) was significantly higher than that of *A. japonicus* (average of 766) (*p* < 0.01) (Figure 6).

### 3.4. Venom Protein Identification and Functional Analysis

**Figure 7:**
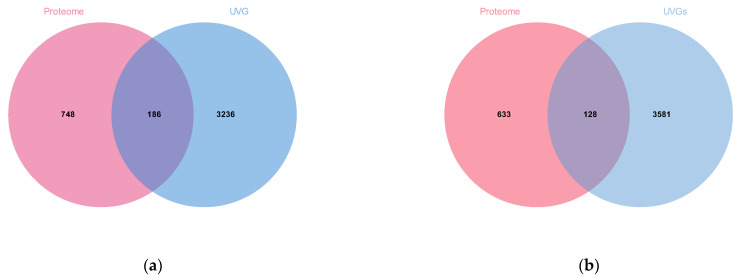
Venom diagram of putative venom proteins of two parasitoids combining transcriptomic and proteomic analysis. (**a**) The intersection of UVGs and those matched to the proteomic databases from *Mesocomys trabalae*. (**b**) The intersection of UVGs and those matched to the proteomic databases from *Anastatus japonicus*.

Venom proteins of *M. trabalae* and *A. japonicus* were identified by a combined venom gland transcriptome and venom proteome approach. The 3422 UVGs of *M. trabalae* had 186 proteins identified in the proteome (Figure 7a). The functions of these proteins are listed, and 179 potential venom proteins are known, such as lipid transport and metabolism, amino acid transport and metabolism, and signal transduction mechanisms. Meanwhile, there are 7 proteins with unknown functions (Table S1). The 3709 UVGs of *A. japonicus* had 128 proteins identified in the proteome (Figure 7b); the functions of these proteins were annotated, and 98 of them were known, such as lipid transport and metabolism, carbohydrate transport and metabolism, and amino acid transport and metabolism. Meanwhile, there were 30 proteins with unknown functions (Table S2).

## 4. Discussion

Parasitoids are essential species in natural ecosystems and can also be used as valuable and environmentally friendly biocontrol agents to control various pests [45]. Their successful parasitism depends on parasitic factors, and venom is one of the important parasitic factors [11,39]. Therefore, it is imperative to understand the venom protein to clarify the parasitic mechanism. Research on the protein composition of egg parasitoid venom is relatively lacking. The existing research has identified more than 10 enzymes from parasitoids venom through enzyme activity determination, gene sequence comparison analysis, and proteome methods, including metalloproteinase, peptidase, serine proteinase, phenol oxidase, trehalase, chitinase, and acid/alkaline phosphatase [46]. These enzymes have different functions in the host [47]. In this study, the residues of *M. trabalae* and *A. japonicus* and the transcriptome of the venom gland were sequenced by high-throughput transcriptome sequencing technology, and the genes highly expressed in the venom gland were screened out. Because the venom protein was synthesized in the venom gland, secreted, and stored in the venom sac, these UVGs may be transcripts of the genes encoding the venom protein or may participate in the expression of the venom proteins; other auxiliary factors may also be translated and assembled. Therefore, we sequenced the venom proteome to identify only the components of the venom protein. The potential venom protein exists in both the transcriptome and the proteome.

With the development of sequencing technology, the identification of parasitoid venom proteins has also made significant progress [29,30,31,45,48,49,50,51]. Despite strict filtration, it is still found that their venom composition is very complex, and the number and species are higher than those of other Hymenoptera insects with venom [19], which may be related to their need to adapt to a variety of hosts [2,52]. The venom of social insects such as ants, bees, spiders, and centipedes, is mainly composed of small molecular peptides [53,54]. The venom proteins of parasitoids are mostly macromolecular proteins [55]. However, parasitic wasps may release different factors when parasitizing different hosts. In this study, the venom proteins of both parasitoid species were also more than 45 kD (Figure 5). The venom proteins of parasitoids vary greatly among distant species [27,31]. Ye found that the genes encoding venom proteins belong to a rapidly evolving group in the genome with rich diversity [38]. In this study, *M. trabalae* and *A. japonicus* are parasitoids of two genera, and the latter has more host species than the former. We assumed that the number of venom proteins would also be more abundant. However, the results were entirely unexpected. Although the number of UVGs (3422) in the *M. trabalae* venom gland was less than that of *A. japonicus* (3709), not only did the proteome get more protein (Figure 6), but the number of potential venom proteins screened by combining the two methods was more than that of *A. japonicus* (Figure 7). But of the 186 venom proteins of *M. trabalae*, 179 are known in function, and only 7 are unknown. However, 30 venom proteins in *A. japonicus* are unknown, and 98 have known functions. It can be speculated that *A. japonicus* may evolve a unique venom protein due to its adaptation to the need to find a host, which is not found in many other parasitoids. Although the quantity of venom protein of *M. trabalae* is dominant, most of it can be identified, so it may be relatively conservative.

Parasitic wasp venom mainly targets immunity, development, and metabolism [56], and sometimes even the host nervous system, to ensure successful parasitism [19,20]. This is very different from the venom function of social Hymenoptera insects, which are mainly used for predation and defense [46]. However, research on its function in egg parasitoids is very scarce, which may be due to the fact that the egg is a relatively fragile period of an insect and its physiological functions are not fully developed, so it is difficult to carry out functional verification, which is also a technical problem to be overcome in the future. When commenting on the GO function of venom proteins in both parasitoid species, we found that the venom proteins mostly belong to the classification of molecular function and biological process and rarely involve the cellular component. The results also conform to the characteristics of venom proteins, most of which have clear biological functions, such as lipid transport and metabolism, amino acid transport and metabolism, and lyase and hydrolase activities (Table S2). We also found that *M. trabalae* venom proteins, relative to those of *A. japonicus,* have a large distribution of functions, especially in molecular function.

The venom proteins of *M. trabalae* and *A. japonicus* have been preliminarily identified. Due to the lack of functional research on the venom protein of egg parasitism, future research will focus on some proteins with special functions. Therefore, the data sets of both parasitoids obtained in this study will provide a theoretical basis for exploring the parasitic mechanism of egg parasitoids and improving the parasitic ability of parasitic wasps to improve the biological control effect in the future.

## 5. Conclusions

In this study, we used a combination of transcriptomic and proteomic approaches to identify the protein fractions of the venom in both eupelmid egg parasitoids, *A. japonicus* and *M. trabalae*. We identified 3422 up-regulated venom gland genes in *M. trabalae* and 3709 in *A. japonicus* and analyzed their functions comparatively. By proteome sequencing, we identified 956 potential venom proteins in the venom pouch of *M. trabalae*, of which 186 were contained in up-regulated genes simultaneously. A total of 766 proteins were detected in the venom of *A. japonicus*, of which 128 venom proteins were highly expressed in the venom glands. The results of functional analysis for these identified venom proteins indicated that the venom proteins in *M. trabalae* are well known, but not in *A. japonicus,* which may be related to the host range.

## Figures and Tables

**Table 1:** Summary of the transcriptomes of *Mesocomys trabalae* and *Anastatus japonicus*.

Species	Samples	Total Number of Reads	Total Number of Bases	Q30 (%)	GC (%)	Total Number of Genes	Percentage Compared to Reference Genome (%)
*M. trabalae*	Mt_vg_1 ^1^	49,726,064	7.46 Gb	92.02	43.93	22,691	94
	Mt_vg_2	47,288,482	7.09 Gb	92.3	49.46		
	Mt_vg_3	49,488,022	7.42 Gb	92.28	44.24		
	Mt_body_1 ^2^	45,211,074	6.78 Gb	92.24	41.87		
	Mt_body_2	44,573,442	6.69 Gb	91.22	42.32		
	Mt_body_3	44,435,666	6.67 Gb	92.12	45.34		
*A. japonicus*	Aj_vg_1 ^3^	45,558,606	6.83 Gb	92.11	41.65	20,860	93
	Aj_vg_2	45,633,086	6.84 Gb	92.26	35.98		
	Aj_vg_3	47,038,826	7.06 Gb	93.68	36.9		
	Aj_body_1 ^4^	48,425,812	7.26 Gb	91.88	35.21		
	Aj_body_2	54,616,318	8.19 Gb	90.98	36.43		
	Aj_body_3	45,866,146	6.88 Gb	91.72	36.22		

Note: ^1^: Mt_vg_1-3: *M. trabalae* venom glands; ^2^: Mt_body_1-3: *M. trabalae* bodies without venom gland; ^3^: Aj_vg_1-3: *A. japonicus* venom glands; ^4^: Aj_body_1-3: *A. japonicus* bodies without venom gland.

## Data Availability

The raw transcriptome data of *M. trabalae* and *A. japonicus* have been uploaded to the NCBI Sequence Read Archive (SRA) database with accession numbers SRR23851092, SRR23851091, SRR23851090, SRR23851089, SRR23851088, SRR23851087, SRR23864786, SRR23864787, SRR23864788, SRR23864789, SRR23864790, SRR23864791 (Bioproject: PRJNA943600). The venom protein data have been uploaded to Integrated Proteome Resources (iProX) in Project ID: IPX0006159000.

## Supplementary Materials

The following supporting information can be downloaded at: https://www.mdpi.com/article/10.3390/biology12050700/s1, Table S1: Function Description of Venom proteins of *Mesocomys trabalae*; Table S2: Function Description of Venom proteins of *Anastatus japonicus*.
